# Higher Dietary Vitamin K Intake is Associated with Better Physical Function and Lower Long-Term Injurious Falls Risk in Community-Dwelling Older Women

**DOI:** 10.1007/s12603-022-1866-9

**Published:** 2023-01-10

**Authors:** Marc Sim, C. Smith, N.P. Bondonno, S. Radavelli-Bagatini, L.C. Blekkenhorst, J. Dalla Via, R. McCormick, K. Zhu, J.M. Hodgson, R.L. Prince, J.R. Lewis

**Affiliations:** 1 Nutrition & Health Innovation Research Institute, School of Health and Medical Sciences, Edith Cowan University, Perth, Western Australia, Australia; 2 Medical School, The University of Western Australia, Perth, Western Australia, Australia; 3 Royal Perth Hospital Research Foundation, Perth, Western Australia, Australia; 4 Institute for Health and Sport (IHES), Victoria University, Victoria, Australia; 5 Australian Institute for Musculoskeletal Science (AIMSS), University of Melbourne and Western Health, Victoria, Australia; 6 Danish Cancer Society, Copenhagen, Denmark; 7 Department of Endocrinology and Diabetes, Sir Charles Gairdner Hospital, Nedlands, Western Australia, Australia; 8 School of Public Health, Curtin University, Perth, Western Australia, Australia; 9 Hospital at Westmead, School of Public Health, Sydney Medical School, The University of Sydney, Sydney, New South Wales, Australia; 10 Nutrition & Health Innovation Research Institute, School of Medical and Health Sciences, Edith Cowan University, 6027, Joondalup, WA, Australia

**Keywords:** Phylloquinone, menaquinone, muscle function, injury, nutrition

## Abstract

**Background:**

In recent years, a potential beneficial role of Vitamin K in neuromuscular function has been recognised. However, the optimal dietary intake of Vitamin K to support muscle function in the context of falls prevention remains unknown.

**Objective:**

To examine the relationship of dietary Vitamin K1 and K2 with muscle function and long-term injurious fall-related hospitalisations in older women.

**Design:**

Cohort study.

**Participants:**

1347 community-dwelling older Australian women ≥70 years.

**Measurements:**

A new Australian Vitamin K nutrient database, supplemented with published data, was used to calculate Vitamin K1 and K2 intake from a validated food frequency questionnaire at baseline (1998). Muscle function (grip strength and timed-up-and-go; TUG) as well plasma Vitamin D status (25OHD) were also assessed at baseline. Fall-related hospitalisations over 14.5 years were obtained from linked health records. Multivariable-adjusted logistic regression and Cox-proportional hazard models were used to analyse the data.

**Results:**

Over 14.5 years of follow-up (14,774 person-years), 535 (39.7%) women experienced a fall-related hospitalisation. Compared to women with the lowest Vitamin K1 intake (Quartile 1, median 49 *µ*g/d), those with the highest intake (Quartile 4, median 120 *µ*g/d) had 29% lower odds (OR 0.71 95%CI 0.52–0.97) for slow TUG performance (>10.2 s), and 26% lower relative hazards of a fall-related hospitalisation (HR 0.74 95%CI 0.59–0.93) after multivariable adjustment. These associations were non-linear and plateaued at moderate intakes of ∼70–100 *µ*g/d. There was no relation to grip strength. Vitamin K2 intakes were not associated with muscle function or falls.

**Conclusion:**

A higher habitual Vitamin K1 intake was associated with better physical function and lower long-term injurious falls risk in community-dwelling older women. In the context of musculoskeletal health, Vitamin K1 found abundantly in green leafy vegetables should be promoted.

## Introduction

**F**alls and associated injuries are a frequent and major public health concern in older populations. One in three adults over 65 years will suffer a fall each year (1). Unsurprisingly, falls are the most common cause of hospitalised injury in this age group (2). In addition to injury such as hip fracture, laceration, concussion and trauma of falling, the fear of falling again often leads to reduced social engagement and avoiding physical activity (3). These can lead to permanent mobility limitations that compromise independence and quality of life (1). Consequently, falls can have detrimental long-term physical and mental health impact, contributing to chronic disease burden (4). This is especially concerning for older women who are particularly susceptible to osteoporosis and have up to 60% greater risk for sustaining an injurious fall compared to their male counterparts (2, 5).

Previous dietary studies have typically focused on protein, calcium, and Vitamin D supplements to improve muscle function for falls prevention, with mixed results to date (6). Recent work suggests that Vitamin K could play a role in muscle and bone metabolism potentially through the carboxylation of the Vitamin K dependant proteins, including osteocalcin (OC) and matrix Gla protein (7). In support of this concept, we previously reported that poorer Vitamin K status in older women, indicated by a higher ratio of undercarboxylated OC to total OC (ucOC:tOC), was associated with poorer physical function and greater falls risk (8). Others have also reported that lower Vitamin K status (desphospho-uncarboxylated matrix Gla protein; de-ucMGP) in older adults is associated with compromised muscle function (9) and greater frailty risk over 13 years (10). Whilst such studies suggest Vitamin K status has the potential to lower falls risk, the optimal dietary intake of Vitamin K to support such benefits remains unknown.

Dietary Vitamin K consists of two main forms, Vitamin K1 (phylloquinone, PK) and K2 (menaquinone, MK). Green leafy vegetables are a rich source of Vitamin K1, while animal products including fermented foods, yoghurt, milk and cheese are abundant in Vitamin K2 (11). It is reported that the bioavailability and pharmokinetics vary between Vitamin K1 and K2, potentially due to enzyme affinity and tissue distribution (12). Taken in consideration with the dietary sources of Vitamin K1 and K2, this warrants separate examination. Nutrition guidelines from the National Institute of Health (13) and the International Osteoporosis Foundation (14) also highlight uncertainty regarding the benefits of Vitamin K for musculoskeletal health. Therefore, the aim of this study was to determine if higher dietary Vitamin K1 and/or K2 intake is associated with better muscle function and lower risk for injurious falls in community-dwelling older women. Furthermore, we sought to examine whether dose-dependent thresholds existed for associations with injurious falls risk.

## Methods

### Participants

The Calcium Intake Fracture Outcome Study (CAIFOS) study is a 5-year, double-blind, randomised controlled trial of daily calcium supplementation to prevent fractures in older women that commenced in 1998. Women (n=1500, aged ≥70) with (i) an expected survival beyond 5 years and (ii) not receiving any medication (including hormone replacement therapy) known to affect bone metabolism (15) were recruited using electoral roll. Women were invited to be part of 10 years of observational follow-up (two studies of five years each), leading to a total follow-up of 14.5 years; the Perth Longitudinal Study of Aging Women (PLSAW). At baseline, 1485 women completed a food frequency questionnaire (FFQ). Individuals with implausible energy intakes (<2100 kJ [500 kcal] or >14,700 kJ [3500 kcal]; n=17/1485, 1.1%)(16) or undertaking Vitamin D supplementation (due to its link with falls; n=39/1485, 2.6%) were excluded. Women taking warfarin (n=45) were also excluded due to its known interference with Vitamin K metabolism (17). Individuals who had missing data for any covariate were also excluded (n=37). The current study included 1347 women (Supplementary Figure 1). Of note, 98 of the 1347 women did not have Vitamin D status (25OHD) measured. As such, any analysis where 25OHD was included as a covariate had a sample size of 1249 women. For all individuals, written informed consent was obtained. The Human Research Ethics Committee of the University of Western Australia provided ethical approval. Both CAIFOS and PLSAW complied with the Declaration of Helsinki and were retrospectively registered on the Australian New Zealand Clinical Trials Registry (#ACTRN12615000750583 and #ACTRN12617000640303). Linked data ethics approval was provided by the Human Research Ethics Committee of the Western Australian Department of Health (#2009/24). STROBE-NUT guidelines for observational studies were adhered to for this current manuscript.

**Figure 1:**
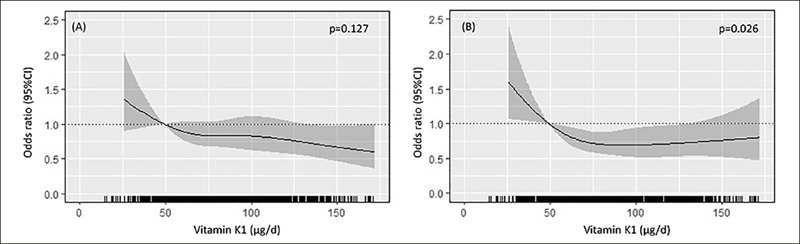
Odds ratios from multivariable-adjusted logistic regression models with restricted cubic spline curves describing the association between Vitamin K1 and (A) weak hand grip strength; <22 kg, and (B) slow-timed-up-and-go; >10.2 s

Odds ratios are based on models adjusted for age, treatment, BMI, smoking history, prevalent falls, alcohol intake and physical activity (Model 2). The odds ratio compares the specific intake of Vitamin K1 (horizontal axis) to the median intake in the lowest quartile (49 µg/d). Shading represents 95% confidence regions. The rug plot along the bottom of each graph depicts each observation

### Dietary intake

A self-administered, semiquantitative FFQ developed and validated by the Cancer Council of Victoria was used to determine dietary intake at baseline in 1998 (18, 19). The FFQ was designed to capture diet over a year, with such timeframes used to represent habitual intake. Participants were provided with measuring spoons and cups, in conjunction with food models and charts while completing the FFQ. A research assistant also supported these women when completing the FFQ to enhance the accuracy of reported food consumption. Nutrient intakes including calcium (mg/d), alcohol (g/d) and protein (g/d) were calculated using the NUTTAB95 food composition database (20). Where necessary, other sources were considered (21). Overall diet quality was assessed via the Nutrient Rich Foods Index standardised per 1000 kJ of energy intake (NRFI, described previously (22)).

The PK and MK values of commonly consumed foods from an Australian food database specific to Vitamin K were obtained for each food item (11). Vitamin K2 comprises of isoprenologs with varying number of isoprenoid units ranging from four to thirteen repeats (MK4 to MK13) (17). Approximately 10% of total Vitamin K intake is estimated to come from Vitamin K2, with up to 40% attributed to MK4 (23, 24). From the 101 foods and beverages (including alcohol) obtained from the FFQ, the Australian Vitamin K nutrient database assessed 56 food items known to contain PK, MK4, or MK7. For this database, the main food groups included vegetables (n=20), fruit (n=3), animal products (n=16), dairy (n=14) and fermented foods (n=3). Where the PK content of the FFQ items were not quantified by this Australian Vitamin K nutrient database (n=45), values were obtained from the United States Department of Agriculture (USDA) Food and Nutrient Database for Dietary Studies 2017–18 (25). Where the MK4 to MK10 content of the FFQ items were not quantified or available from the Australian Vitamin K nutrient database, values were obtained from Schurgers et al. (23) (MK4, MK5, MK6, MK7, MK8 and MK9), and Manoury et al. (27) (MK10). As such, Vitamin K2 intake included MK4 to MK-10. Intake of PK and MK from all food and beverage items (n=101) included on the FFQ were obtained by multiplying the food item consumed (g/d) by the estimated Vitamin K (PK, MK4 to MK10) content (*µ*g/g), similar to previous work (26). Upon reasonable request to the corresponding author, data on the values used and assumptions made can be provided.

### Muscle function

Grip strength, which measures the amount of force (kg) the forearm flexors can produce using a dynamometer (Jamar Hand Dynamometer, Lafayette Instrument Company, USA), was used to quantify upper-limb muscular strength. One practice and three test trials were performed using participants' dominant hand, with the peak value recorded. TUG measures the time it takes an individual to rise from a chair (46 cm seat height), walk 3 m, turn around, and return to sit on the chair. Participants performed one practice trial before undertaking a recorded trial. TUG is commonly adopted method to assess functional mobility among older adults in geriatric clinics to evaluate physical performance (28). The inter-observer CV error was 7% for hand grip strength and 6% for TUG in our laboratory as assessed on a random sample of 30 subjects. Cut-points for weak grip strength (<22 kg) and slow TUG (>10.2 s) were selected based on previous work reporting clinical muscle weakness (29) and compromised physical function (30).

### Fall-related hospitalisations

Linked falls-related hospitalisations over 14.5 years were drawn from the Western Australia Hospital Morbidity Data Collection (HMDC), via the Western Australian Data Linkage System (Department of Health Western Australia, East Perth, Australia). Falls were considered injurious if they were serious enough to require hospitalisation. HMDC records were obtained for each of the study participants from the date of their clinical visit in 1998. Falls-related hospitalisations were identified using the international classification of external causes of injury codes and the International Classification of Diseases (ICD) coded discharge data pertaining to all public and private inpatient admissions in Western Australia. This allows ascertainment of hospitalisations independent of self-report and avoids the problems of patient self-reporting and loss to follow-up. As described previously (31), falls from standing height or less, not resulting from external force were included (ICD-10 codes W01, W05–W08, W10, W18, and W19). Where ICD-10 coded death data were not yet available for a participant, we used Parts 1 and 2 of the death certificate or all diagnosis text fields from the death certificate.

### Baseline characteristics

Physical activity and smoking history were obtained via questionnaire. Participation in sport, recreation, and/or regular physical activities undertaken in the 3 months prior to participants baseline visit was examined with energy expenditure (kJ/d) calculated by considering activity type, time undertaken and body weight (3). An individual was considered an ex-smoker/current smoker if they had consumed >1 cigarette per day for more than 3 months at any time in their life. Digital scales were used to assess body weight, while height was obtained using a stadiometer. These measures were used to calculate body mass index (BMI, kg/m^2^). CAIFOS treatment code (placebo or calcium) was included as a covariate. Self-reported prevalent falls in the 3 months prior to the baseline clinical visit were captured via questionnaire.

All women had venous blood samples obtained in the morning after an overnight fast (0830 to 1030 h) at their baseline clinic visit. Samples were subsequently stored at −80°C until analysis. A validated LC-MS/MS (Liquid Chromatography Tandem Mass Spectrometry) method adopted at the RDDT Laboratories (Bundoora, VIC, Australia) was used to measure Vitamin D status via plasma 25-hydroxyVitamin D2 (25OHD2) and D3 (25OHD3) (32). Values were summed to obtain total plasma 25OHD concentration for each individual. Coefficients of variation (CVs) were 10.1% at a 25OHD2 mean concentration of 12 nmol/L and 11.3% at a 25OHD3 mean concentration of 60 nmol/L. One nmol/L of 25OHD is equivalent to 0.4 ng/mL. For descriptive purposes, the season where the blood sample was obtained (Summer [December to February], Autumn [March to May], Winter [June to August] and Spring [September to November] was subsequently combined into two groups, Summer/Autumn vs. Winter/Spring.

### Statistical analysis

For statistical analyses, Stata (version 14 StataCorp LLC, College Station, Texas, USA), IBM SPSS (version 25.0, IBM Corp., Armonk, NY, USA) and R (version 3.4.2, R Foundation for Statistical Computing, Vienna, Austria) (33) were used. Firstly, we examined associations between Vitamin K intake and measures of physical function (grip strength and TUG), cross-sectionally at baseline. To allow associations to be nonlinear, restricted cubic splines within logistic regression models were used to examine the relationship between Vitamin K intake and binary outcomes including weak grip strength and slow TUG using the ‘rms' R package (34). Odds rations (OR) estimates were relative to a reference value being the median Vitamin K intake of participants in Q1 and were being plotted against the respective outcomes with 95% confidence bands provided. Wald tests were used to obtained p-values. For visual simplicity only, the x-axis was truncated at 3 SD above the mean. When these outcomes were modelled as continuous variables, relationships with Vitamin K were examined using restricted cubic splines as part of generalised linear models with associations presented graphically using the ‘effects' R package (35). Differences in grip strength and TUG between quartiles of Vitamin K intake were assessed using MANCOVA. Cox proportional hazards models were used to investigate the relationship between Vitamin K intakes and fall-related hospitalisations using the ‘rms' R package (34) using the same methodology as described above. Schoenfeld residuals indicated that proportional hazards assumptions were not violated. Three models of adjustment were used for all analyses, (i) Model 1: age, treatment code (placebo/calcium) and BMI; (ii) Model 2: Model 1 plus physical activity (kJ/d), smoking history (yes/no), alcohol intake (g/d) and prevalent self-reported falls (yes/no) and; (iii) Model 3: Model 2 plus 25OHD and season.

### Additional analysis

To examine the robustness of the association between Vitamin K1 and fall-related hospitalisations, we included additional covariates, individually, within Model 3. Covariates included muscle function measures (grip strength, TUG), dietary factors (protein, calcium, overall diet quality [NRFI per 1000 kJ, described previously (22)]) and prevalent atherosclerotic vascular disease (ASVD). Prevalent ASVD (n=154) was determined using primary discharge diagnoses from hospital records over the previous 18-years (1980–1998). These included ischemic heart disease and failure, cerebrovascular disease (excluding haemorrhage) and peripheral arterial disease, as described previously (36). This was undertaken as Vitamin K is reported to positively influence vascular health (17).

## Results

At baseline, the mean ± SD intake for Vitamin K1 and K2, was 83 ± 31 *µ*g/d and 38 ± 17 *µ*g/d, respectively. The mean ± SD age and BMI was 75.1 ± 2.7 y and 27.2 ± 4.8 kg/m^2^, respectively. Table 1 reports the baseline characteristics of the women by quartiles of Vitamin K1 intake. Compared to women with the lowest Vitamin K1 intake (Q1), those with highest K1 intake (Q4) were more physically active, less likely to have suffered a prevalent fall and presented with higher 25OHD levels.

**Table 1:** Baseline characteristics in all participants and by quartiles of Vitamin K1 intake^1^

	**All Participants**	**Quartiles of Vitamin K1 intake**			
		**Quartile 1 <61.6***µ***g/d**	**Quartile 2 61.6 to <79.0***µ***g/d**	**Quartile 3 79.0 to <99.2***µ***g/d**	**Quartile 4 ≥99.2***µ***g/d**
Number	1347	337	337	337	336
Age, years	75.1 ± 2.7	75.3 ± 2.8	74.9 ± 2.7	75.2 ± 2.6	75.1 ± 2.6
Treatment group (calcium), n (%)	679 (50.4)	165 (49.0)	164 (48.7)	173 (51.3)	177 (52.7)
Body mass index (BMI), kg/m^2^	27.2 ± 4.8	27.1 ± 4.9	27.0 ± 4.5	27.5 ± 5.0	27.2 ± 4.6
Smoked ever, yes n (%)	501 (37.2)	136 (40.4)	130 (38.6)	119 (35.3)	116 (34.5)
Physical activity, kJ/day	474 (158–860)	452 (0–859)	478 (171–848)	417 (170–838)	520 (220–914)
Hand grip strength, kg	20.5 ± 4.8	20.0 ± 5.0	20.6 ± 4.4	20.7 ± 4.6	20.5 ± 5.1
Timed-up-and-go, s	9.4 (8.1–11.0)	9.8 (8.4–11.2)	9.2 (8.0–10.9)	9.4 (8.0–11.2)	9.2 (8.0–10.8)
Alcohol intake, g/d	1.9 (0.3–9.9)	1.9 (0.3–9.8)	2.1 (0.3–10.3)	1.7 (0.3–9.3)	1.7 (0.3–9.7)
Prevalent falls, yes n (%)	160 (11.9)	50 (14.8)	43 (12.8)	37 (11.0)	30 (8.9)
25OHD, mmol/L^2^	67.0 ± 28.6	61.6 ± 27.4	68.2 ± 31.7	70.0 ± 27.4	68.0 ± 27.3
Blood sample collection season^2^					
Summer/Autumn, n (%)	310 (24.8)	84 (27.2)	68 (21.7)	80 (25.1)	78 (25.3)
Winter/Spring, n (%)	939 (75.2)	225 (72.8)	245 (78.3)	239 (74.9)	230 (74.7)
Vitamin K1 intake, *µ*g/d	82.7 ± 31.3	47.8 ± 10.0	70.3 ± 5.1	87.9 ± 5.6	125.0 ± 23.8
Vitamin K2 intake, *µ*g/d	37.6 ± 16.7	31.4 ± 14.0	36.2 ± 15.9	39.8 ± 17.4	43.1 ± 17.1

1. Data presented as mean ± SD, median (interquartile range; for non-normally distributed variables) or number n and (%). 2n=1249.

### Vitamin K and muscle function

Higher Vitamin K1 intake was associated with a lower odds of slow TUG performance after multivariable adjustments (Figure 1). This relationship appeared to be non-linear, plateauing at intakes of approximately ≥70 *µ*g/d, corresponding to the median intake of participants in Q2. Compared to women with the lowest Vitamin K1 intake (Q1), women with higher intakes (Q2, Q3, Q4) had a 26%, 30% and 29% lower odds of slow TUG performance (Model 2, Table 2). When 25OHD and season were included as covariates (Model 3), 20%, 26%, and 25% lower odds for slow TUG were recorded for women with moderate to high Vitamin K1 intakes (Q2, Q3 and Q4, respectively) compared to those with the lowest intake (Q1), although statistical significance was slightly attenuated for Q4 (Table 2). While Vitamin K1 intakes appeared to be inversely associated with odds of weaker hand grip strength, this association was not statistically significant after multivariable adjustment (Figure 1 and Table 2). Vitamin K2 was not associated with lower odds for weak hand grip strength or slow TUG in any multivariable-adjusted analysis (Supplementary Figure 3, Supplementary Table 3).

**Table 2:** Odds ratio (95%CI) for weak hand grip strength and slow-timed-up-and go performance by quartiles of Vitamin K1 intake

		**Quartiles for Vitamin K1 intake**^1^			
		**Quartile 1 <61.6***µ***g/d**	**Quartile 2 61.6 to <79.0***µ***g/d**	**Quartile 3 79.0 to <99.2***µ***g/d**	**Quartile 4 >99.2***µ***g/d**
Weak hand grip strength <22 kg	Events, n (%)	224 (65.9)	212 (61.8)	200 (58.3)	191 (56.2)
	Model 1	Ref.	0.83 (0.68–1.02)	0.81 (0.64–1.04)	0.74 (0.55–1.00)
	Model 2	Ref.	0.84 (0.69–1.03)	0.84 (0.65–1.07)	0.78 (0.58–1.05)
	Model 3^2^	Ref.	0.83 (0.67-1.03)	0.83 (0.64-1.07)	0.78 (0.57-1.06)
Slow timed-up-and-go >10.2 s	Events, n (%)	149 (43.6)	111 (32.4)	124 (36.2)	114 (33.3)
	Model 1	Ref.	**0.73 (0.60–0.89)**	**0.68 (0.54–0.88)**	**0.70 (0.51–0.95)**
	Model 2	Ref.	**0.74 (0.60–0.90)**	**0.70 (0.54–0.89)**	**0.71 (0.52–0.97)**
	Model 3^2^	Ref.	**0.80 (0.65–0.99)**	**0.74 (0.57–0.96)**	0.75 (0.54–1.04)

1. Estimated odds and 95%CI from logistic regression analysis comparing the median Vitamin K1 intake from each quartile (Q) compared to Q1. Median intake Q1, Q2, Q3 and Q4 for Vitamin K1 was 49.4, 70.2, 87.6 and 119.6 *µ*g/d, respectively. Model 1: adjusted for age, treatment (calcium/placebo) and body mass index. Model 2: Model 1 + smoking history, physical activity and alcohol intake. 2. n=1249. Model 3: Model 2 + season, 25OHD. Bolded indicates p<0.05 compared to Q1.

**Table 3:** Hazard ratios (95%CI) for fall-related hospitalisations over 14.5 years by quartiles of Vitamin K1 and K2 intake

	**Quartiles of Vitamin K1**^1^			
	**Quartile 1 <61.6***µ***g/d**	**Quartile 2 61.6 to <79.0***µ***g/d**	**Quartile 3 79.0 to <99.2***µ***g/d**	**Quartile 4 ≥99.2***µ***g/d**
14.5 y fall-related hospitalisation				
Events, n (%)	148 (43.9)	133 (39.5)	133 (39.6)	121 (35.9)
Model 1	Ref.	**0.84 (0.73–0.96)**	**0.76 (0.64–0.91)**	**0.72 (0.57–0.90)**
Model 2	Ref.	**0.84 (0.73–0.97)**	**0.78 (0.65–0.93)**	**0.74 (0.59–0.93)**
Model 3^2^	Ref.	0.86 (0.74–1.00)	**0.78 (0.64–0.93)**	**0.73 (0.58–0.93)**
	**Quartiles of Vitamin K2**^1^			
	**Quartile 1 <26.2***µ***g/d**	**Quartile 2 26.2 to <35.6***µ***g/d**	**Quartile 3 35.6 to <46.1***µ***g/d**	**Quartile 4 >46.1***µg***/d**
Events, n (%)	129 (38.3)	146 (43.3)	126 (37.5)	134 (39.8)
Model 1	Ref.	1.00 (0.86–1.17)	1.06 (0.88–1.27)	1.07 (0.85–1.34)
Model 2	Ref.	1.00 (0.85–1.16)	1.06 (0.89–1.28)	1.09 (0.87–1.37)
Model 3^2^	Ref.	0.94 (0.81–1.10)	1.05 (0.87–1.27)	1.11 (0.87–1.41)

1. Estimated hazard and 95%CI from Cox proportional hazards analysis comparing the median Vitamin K1 and K2 intake from each quartile (Q) compared to Q1. 2. n=1249. Median Vitamin K1 intake for Q1, Q2, Q3 and Q4 was 49.3, 70.1, 87.6 and 119.5 *µ*g/d, respectively. Median Vitamin K2 intake for Q1, Q2, Q3 and Q4 was 20.2, 31.0, 40.2 and 56.0 *µ*g/d, respectively. Model 1: adjusted for age, treatment and body mass index. Model 2: Model 1 + smoking history, physical activity, prevalent falls, alcohol intake. Model 3: Model 2 + 25OHD and season (Winter/Spring, Summer/Autumn). Bolded indicates p<0.05 compared to Q1.

Visual representations of multivariable-adjusted relationships of Vitamin K1 and K2 intakes with both grip strength and TUG, modelled as continuous variables, are presented in Supplementary Figure 2. Grip strength was not significantly different between quartiles of Vitamin K1 and K2 intake (Supplementary Table 2). For TUG, women with moderate to high Vitamin K1 intakes (Q2, Q3, Q4) were approximately 0.7 s faster compared to those with the lowest intake (Q1) (p=0.007). Vitamin K2 was not associated with TUG.

**Figure 2:**
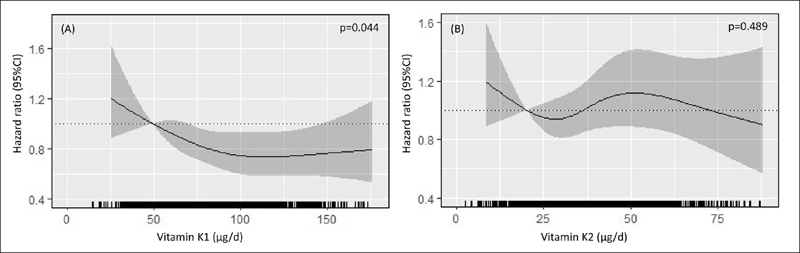
Hazard ratios from Cox proportional hazards model with restricted cubic spline curves describing the association between Vitamin K1 (A) and K2 (B) with fall-related hospitalisations over 14.5 years

Hazard ratios are based on models adjusted age, treatment, BMI, smoking history, physical activity, alcohol intake, prevalent falls, plasma 25OHD and season in 1249 women (Model 3). The hazard ratio compares the specific intake of Vitamin K1 and K2 (horizontal axis) to the median intake in the lowest quartile (49 *µ*g/d and 20 *µ*g/d, respectively). Shading represents 95% confidence regions. The rug plot along the bottom of each graph depicts each observation.

### Injurious fall-related hospitalisation

Of the 1349 women included in the study, 535 (39.7%) experienced a fall-related hospitalisation over 14.5 years (14,774 person-years) of follow-up (mean ± SD, 11.0 ± 4.1 years). Women with the lowest Vitamin K1 intake (Q1) experienced more falls compared to those with the highest Vitamin K1 intake (Q4) (148 vs. 121) (Table 3). In the multivariable-adjusted analysis (Model 2), compared to women with the lowest Vitamin K1 intake, those with moderate to high intakes (Q2, Q3, Q4) had 16% to 26% lower relative hazards for a fall-related hospitalisation (Table 3). When 25OHD status and season were included as covariates in the multivariable-adjusted model (Model 3), these results remained similar (Figure 2, Table 3). There appeared to be a plateau in the relative hazard for a fall-related hospitalisation once Vitamin K1 intakes of approximately 100 *µ*g/d were achieved (Figure 2). For all the aforementioned analyses considering fall-related hospitalisations, no relationship was observed with Vitamin K2 intake (Table 3, Figure 2).

### Additional analyses

The individual inclusion of additional covariates such as grip strength, TUG, prevalent ASVD, dietary protein and calcium intake as well as overall diet quality (NRFI per 1000 kJ) separately to the multivariable-adjusted analysis (Model 3) did not alter the relationship between Vitamin K1 and falls. Compared to women with the lowest Vitamin K1 intake (Q1), those with the higher intakes (Q3 and Q4) continued to consistently record lower relative hazards for a fall-related hospitalisation (Supplementary Table 3).

## Discussion

Our results suggest that higher dietary intake of Vitamin K1, but not Vitamin K2, is associated with better physical function and lower long-term risk for injurious fall-related hospitalisations in community-dwelling older women. Specifically, moderate Vitamin K1 intakes of ∼70 *µ*g/d appears to support some aspects of muscle function such as TUG but not others such as grip strength. Importantly, there is a strong relationship with lower risk for an injurious fall. Specifically, Vitamin K1 intakes of ≥100 *µ*g/d do not appear to further reduce falls risk consistent with a threshold requirement. Notably, the relationship between Vitamin K1 and injurious falls is robust to numerous other risk factors such as plasma 25OHD, muscle function, prevalent ASVD as well as diet quality.

Regarding possible mechanisms for the observed associations, previous cross-sectional studies have also reported a relationship between low Vitamin K status and compromised muscle function (9, 37, 38). For example, in 1089 community-dwelling older adults (mean age 74 years, 67% female), better Vitamin K status (assessed by dp-ucMGP and plasma PK) was associated with better scores on the Short Physical Performance Battery (SPPB), faster 20 m gait speed and higher isokinetic leg strength (37). Individuals with a plasma PK of ≥1.0 nM (indicating better Vitamin K status) also presented with higher SPPB scores and faster 20 m gait-speed 4–5 years later (37). Similar findings have been reported in 633 community-dwelling adults (mean age 60 years, 54% female) from The Longitudinal Aging Study Amsterdam (9). Here individuals with lower Vitamin K status, assessed by higher dp-ucMGP, had poorer grip strength and functional performance. Although dietary intake of Vitamin K was not assessed, these studies suggest an important role for Vitamin K and muscle function.

Notably, when considering poor physical function, a known risk factor for falls (31), women with the highest Vitamin K1 intake had up to 29% lower risk for poor mobility (slow TUG performance). This is supported by previous work from our group reporting that poorer Vitamin K status, detected by higher ucOC:tOC, was associated with compromised physical function, but not muscle strength (8). Given previous data this finding is surprising when considering the added complexity of the TUG test compared to hand grip strength in terms of neuromuscular coordination and balance. It has been suggested that Vitamin K insufficiency can affect calcium metabolism and exacerbate vascular calcium deposition (7). As such, long-term Vitamin K insufficiency could impair neuromuscular coordination (e.g. contraction capacity) and vascular function (e.g. altered blood flow/perfusion at the skeletal muscle), thereby impacting physical function. When considering Vitamin K2, no evidence for a beneficial relationship with muscle function has been reported in older adults (39, 40). For example, a double-blind randomised controlled trial reported that supplementing 200 *µ*g/d or 400 *µ*g/d of pharmaceutical Vitamin K2 (MK7) for 12 months did not affect postural sway and physical function tests (SPBB, TUG and Berg balance scale) in 95 community-dwelling older adults (61% female, mean age 75 years) (40). Collectively, our data suggests that dietary Vitamin K1 (but not Vitamin K2) supports physical function, which is important in the context of falls prevention.

Despite nutrition being regarded as a cornerstone for healthy ageing, dietary guidelines for falls prevention remain elusive; apart from interest in protein and Vitamin D (6). Here, we demonstrate that a minimum Vitamin K1 intake of ∼100 *µ*g/d (lower bound of Q4) would be beneficial in lowering the risk for long-term injurious falls. Such findings support previous work reporting an association between better Vitamin K status (e.g. lower ucOC:tOC) and lower long-term injurious fall risk (8). Considering that vegetables, specifically green leafy and cruciferous varieties, are rich sources of Vitamin K1, our results support benefits of a vegetable-rich diet for musculoskeletal health and falls prevention (41). For Vitamin K2, we found no evidence for a relationship with injurious falls, an observation comparable to previous work supplementing pharmaceutical Vitamin K2 (40).

Based on estimated median intakes, Nutrient Reference Values in Australia for total Vitamin K intake are 70 and 60 *µ*g/d for men and women, respectively (42). Current dietary recommendations for Vitamin K do not differentiate between Vitamin K1 or K2. This is further complicated as the dietary intake of Vitamin K in Australia have been estimated using only international food databases, with the Vitamin K content of food known to vary by up to 50% by region (43). In the context of our findings, such low intakes would not be sufficient to optimise physical function and lower injurious falls risk in women, where only 60 *µ*g/d of dietary Vitamin K is currently recommended for such populations in Australia (42). We have also reported in a randomised controlled trial that such low Vitamin K intakes would be inadequate to support optimal bone metabolism (44). Alternatively, the higher intakes for total Vitamin K promoted in the United States of 120 and 90 *µ*g/d for older men and women, respectively, would be preferable. Most importantly, Vitamin K1 intakes of 100 *µ*g/d or more can easily be attained by consuming 75 g to 150 g (one to two serves) per day of vegetables such as cabbage, rocket (arugula), lettuce, broccoli and spinach (11). Another factor that should be considered are the dietary sources of Vitamin K1 and K2. For example, a diet rich in vegetables providing Vitamin K1 often forms the foundation for a healthy diet, as opposed to animal-based products providing Vitamin K2, habitually consumed in excess. In light of these findings, from a public health perspective, promoting vegetable consumption to ensure adequate Vitamin K nutrition should be promoted.

Despite these promising results, our study does present with limitations. Firstly, the observational nature of this work does not allow for causality to be established. Observational work can also be affected by residual confounding. To minimise this, we considered various risk factors including plasma 25OHD, prevalent ASVD as well as individual dietary factors (protein, alcohol, calcium) and overall diet quality (NRFI per 1000 kJ). Our results remain largely unchanged. Dietary intake obtained from the FFQ was also self-reported and may be subject to misclassification and/or recall bias. However, we adopted both a reproducible and validated method to assess dietary intake. Further, despite assessing the longitudinal relationship between Vitamin K and falls, when considering muscle function, this was limited to cross-sectional data. Finally, results may only be limited to older community-dwelling women and not other groups such as their male counterparts. Nevertheless, older women have the greatest risk for injurious falls and resulting fractures (2, 5). Alternatively, our work does present with numerous strengths. This includes the prospective design and population-based setting with verified falls-related hospitalisations (independent of self-report) from linked health records over 14.5 years. These injurious falls represent the most serious type of falls. We also calculated dietary Vitamin K intake based on a database that measured the Vitamin K content of foods in Australia, which we have since correlated with ucOC:tOC, a biomarker of Vitamin K status, in this cohort (26). This is important as the Vitamin K content of food can vary based on geographical location (17). Finally, we adopted standardised muscle function tests (grip strength, TUG) which are highly relevant to other musculoskeletal diseases including sarcopenia and osteoporosis.

In summary, our findings suggest that moderate Vitamin K1 intakes ∼100 *µ*g/d (e.g. 75–150 g or 1–2 servings of cabbage, rocket, lettuce, broccoli, or spinach per day) may positively influence physical function and reduce long-term risk for injurious falls in community-dwelling older women. For Vitamin K2, no such benefits were observed. Public health guidelines should continue to promote higher vegetable intake, including the daily consumption of Vitamin K1-rich green leafy vegetables to optimise musculoskeletal health.

## Electronic supplementary material

Supplementary material, approximately 474 KB.

*Author contributions:* MS, LCB, JMH, RLP, JRL designed the research. KZ, RLP, JRL conducted the research. MS, CS, RM, LCB, SRB, NPB analysed the data. MS, CS, JDL, RLP, JRL wrote the paper. MS has primary responsibility for the final content. All authors read and approved the final manuscript.

*Funding Statement:* The Perth Longitudinal Study of Ageing in Women (PLSAW) was funded by Healthway, the Western Australian Health Promotion Foundation and by project grants 254627, 303169 and 572604 from the National Health and Medical Research Council (NHMRC) of Australia. This work was supported by a Department of Health, Western Australia, Merit Award. MS is supported by a Royal Perth Hospital Career Advancement Fellowship (CAF 130/2020), an Emerging Leader Fellowship and project grant from the Western Australian Future Health and Innovation Fund, Department of Health (WA). LCB is supported by a National Health and Medical Research Council (NHMRC) of Australia Emerging Leadership Investigator Grant (ID: 1172987) and a National Heart Foundation of Australia Post-Doctoral Research Fellowship (ID: 102498). JMH is supported by an NHMRC of Australia Senior Research Fellowship (ID: 1116973). JRL is supported by a National Heart Foundation of Australia Future Leader Fellowship (ID: 102817). None of these funding agencies had any role in the conduct of the study; collection, management, analysis or interpretation of the data; or preparation, review or approval of the manuscript. Open Access funding enabled and organized by CAUL and its Member Institutions.

*Disclaimers:* All authors declare no conflicts of interest.
