# Supplementary material for: Higher Dietary Vitamin K Intake is Associated with Better Physical Function and Lower Long-Term Injurious Falls Risk in Community-Dwelling Older Women
Source: J Nutr Health Aging. 2023 Jan 10;27(1):38–45. doi: 10.1007/s12603-022-1866-9 (PMC12880029; doi:10.1007/s12603-022-1866-9)
Supplement: Supplementary file 1 — Supplementary material, approximately 474 KB. [file mmc1.docx]

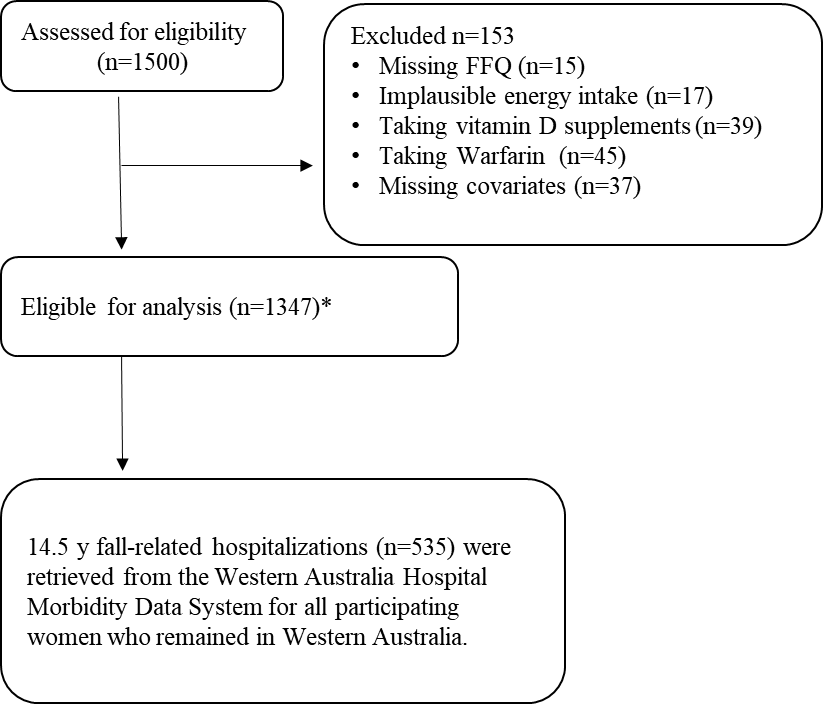


**Supplementary Figure 1.** Participant flow chart. * 25OHD not measured in 98 of the 1347 women (n=1249).

**
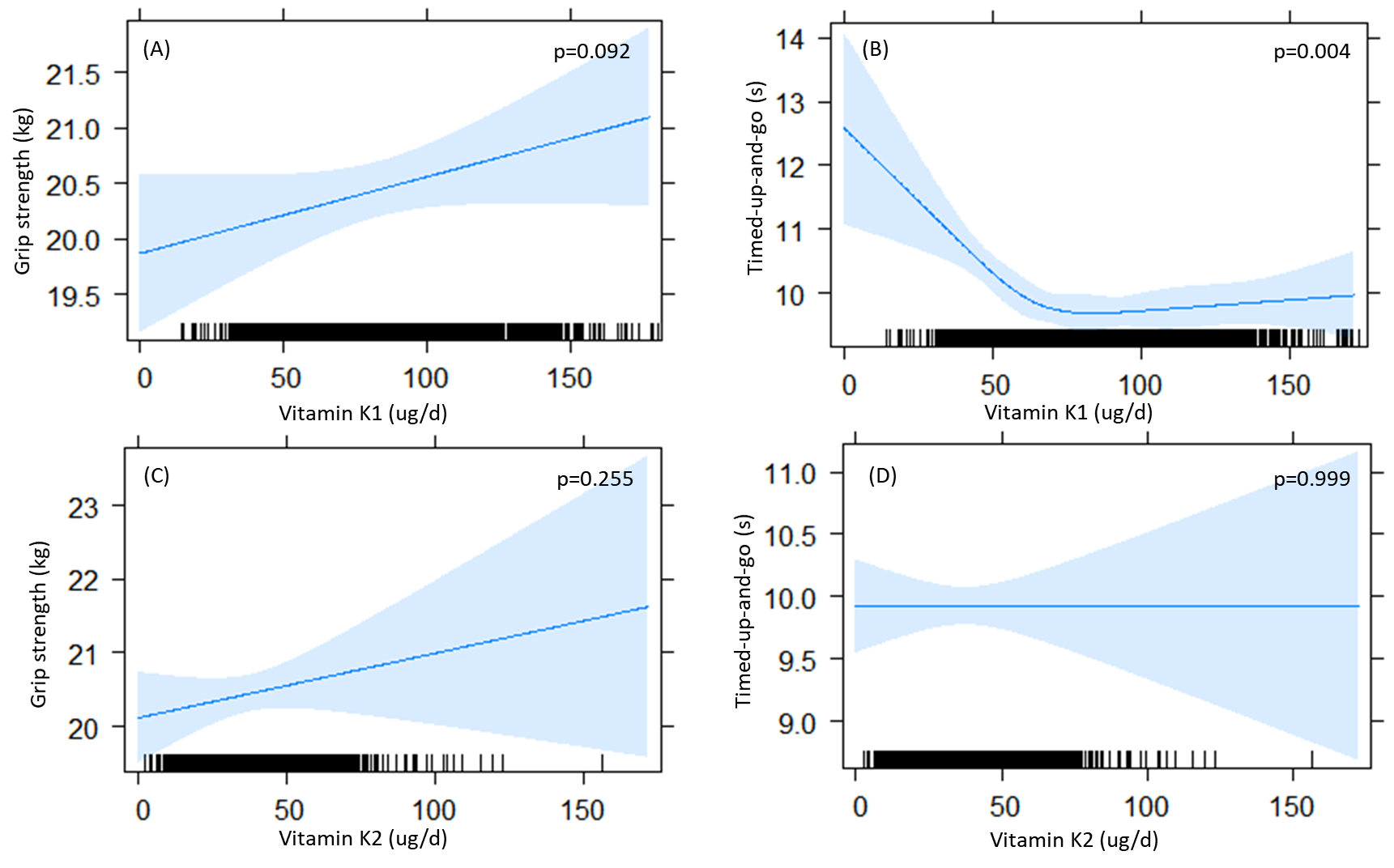
**

**Supplementary Figure 2.** Multivariable-adjusted relationship between Vitamin K1 (A, B) and K2 (C, D), with hand grip strength and the timed-up-and-go test obtained by generalized regression models in 1347 women. Shading represents 95% confidence intervals. The rug plot along the bottom of each graph depicts each observation. Multivariable-adjusted model included age, treatment (calcium/placebo) body mass index, smoking history, prevalent falls, alcohol intake and physical activity.


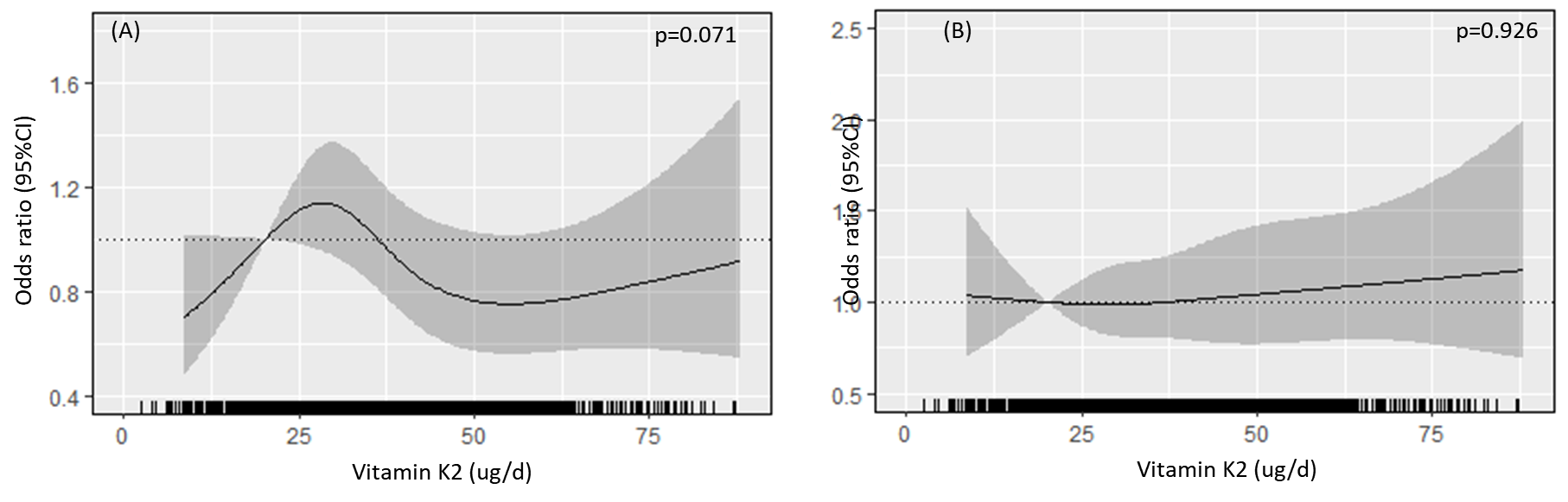


**Supplementary Figure 3.** Odds ratios from multivariable-adjusted logistic regression models with restricted cubic spline curves describing the association between Vitamin K2 and (A) weak hand grip strength; <22 kg, and (B) slow-timed-up-and-go; >10.2 s. Odds ratios are based on models adjusted age, treatment, BMI, smoking history, prevalent falls, alcohol intake and physical activity. The odds ratio compares the specific intake of Vitamin K2 (horizontal axis) to the median intake in the lowest quartile (20 ug/d). Shading represents 95% confidence regions. The rug plot along the bottom of each graph depicts each observation.

**Supplementary Table 2.** Estimated marginal means (standard error) for hand grip strength and timed-up-and-go for each quartile of Vitamin K1 and K2 intake.

|  |  | **Quartiles for Vitamin K1 intake ^1^** | | | |
| --- | --- | --- | --- | --- | --- |
|  |  | **Quartile 1**  <61.6 ug/d | **Quartile 2**  61.6 to <79.0 ug/d | **Quartile 3**  79.0 to <99.2 ug/d | **Quartile 4**  ≥99.2 ug/d |
| *Grip strength (kg)* | *Model 1* | 20.1 (0.3) | 20.5 (0.3) | 20.7 (0.3) | 20.5 (0.3) |
|  | *Model 2* | 20.1 (0.3) | 20.6 (0.3) | 20.7 (0.3) | 20.5 (0.3) |
|  | *Model 3* | 20.1 (0.3) | 20.6 (0.3) | 20.7 (0.3) | 20.4 (0.3) |
| *Timed-up-and-go (s)* | *Model 1* | 10.5 (0.2) | **9.8 (0.2)** | **9.7 (0.2)** | **9.7 (0.2)** |
|  | *Model 2* | 10.4 (0.2) | **9.7 (0.2)** | **9.7 (0.2)** | **9.7 (0.2)** |
|  | *Model 3* | 10.4 (0.2) | **9.7 (0.2)** | **9.8 (0.2)** | **9.8 (0.2)** |
|  |  | **Quartiles for Vitamin K2 intake ^1^** | | | |
|  |  | **Quartile 1**  <26.2 ug/d | **Quartile 2**  26.2 to <35.6 ug/d | **Quartile 3**  35.6 to <46.1 ug/d | **Quartile 4**  ≥46.1 ug/d |
| *Grip strength (kg)* | *Model 1* | 20.3 (0.3) | 20.2 (0.3) | 20.7 (0.3) | 20.6 (0.3) |
|  | *Model 2* | 20.3 (0.3) | 20.2 (0.3) | 20.7 (0.3) | 20.6 (0.3) |
|  | *Model 3* | 20.2 (0.3) | 20.2 (0.3) | 20.7 (0.3) | 20.7 (0.3) |
| *Timed-up-and-go (s)* | *Model 1* | 10.0 (0.2) | 10.0 (0.2) | 9.8 (0.2) | 9.9 (0.2) |
|  | *Model 2* | 10.0 (0.2) | 9.9 (0.2) | 9.9 (0.2) | 9.9 (0.2) |
|  | *Model 3* | 10.0 (0.2) | 9.9 (0.2) | 9.8 (0.2) | 9.9 (0.2) |

^1^ Means (standard error) obtained from generalised linear model. Bolded indicates p≤0.05 compared to Quartile 1. Model 1: adjusted for age, treatment (calcium/placebo) and body mass index. Model 2: Model 1 + smoking history, prevalent falls, alcohol intake and physical activity. Model 3: Model 2 + season, 25OHD.

**Supplementary Table 3.** Odds ratio (95%CI) for weak hand grip strength and slow-timed-up-and go performance by quartiles of Vitamin K2 intake.

|  |  | **Quartiles for Vitamin K2 intake ^1^** | | | |
| --- | --- | --- | --- | --- | --- |
|  |  | **Quartile 1**  <26.2 ug/d | **Quartile 2**  26.2 to <35.6 ug/d | **Quartile 3**  35.6 to <46.1 ug/d | **Quartile 4**  ≥46.1 ug/d |
| *Weak hand grip strength*  *<22 kg* | *Events, n(%)* | 207 (61.2) | 222 (66.3) | 199 (59.1) | 190 (56.4) |
|  | *Model 1* | Ref. | 1.12 (0.93-1.37) | 0.89 (0.70-1.12) | **0.73 (0.55-0.98)** |
|  | *Model 2* | Ref. | 1.12 (0.92-1.37) | 0.90 (0.71-1.13) | 0.75 (0.56-1.01) |
|  | *Model 3* | Ref. | 1.13 (0.92-1.39) | 0.89 (0.69-1.13) | 0.75 (0.55-1.02) |
| *Slow timed-up-and-go*  *>10.2 s* | *Events, n(%)* | 115 (34.0) | 127 (37.9) | 118 (35.0) | 128 (38.0) |
|  | *Model 1* | Ref. | 0.97 (0.79-1.19) | 0.97 (0.76-1.23) | 1.00 (0.74-1.37) |
|  | *Model 2* | Ref. | 0.99 (0.81-1.21) | 1.01 (0.79-1.29) | 1.06 (0.78-1.45) |
|  | *Model 3* | Ref. | 0.97 (0.78-1.20) | 1.02 (0.79-1.32) | 1.10 (0.79-1.53) |

^1^Estimated odds and 95%CI from logistic regression analysis comparing the median Vitamin K1 intake from each quartile (Q) compared to Q1. Median intake Q1, Q2, Q3 and Q4 for Vitamin K2 was 20.2, 31.0, 40.2 and 56.0 ug/d, respectively. Bolded indicates p<0.05 compared to Q1. Model 1: adjusted for age, treatment (calcium/placebo) and body mass index. Model 2: Model 1 + smoking history, prevalent falls, alcohol intake, physical activity and alcohol intake. Model 3: Model 3: Model 2 + season, 25OHD.

**Supplementary Table 4.** Hazard ratios (95%CI) for fall-related hospitalisations over 14.5 years by quartiles of Vitamin K1 intake after individually adjusting for hand grip strength, timed-up-and go performance, diet quality using the Nutrient Rich Food Index (NRFI, standardised per 1000 kJ of energy intake) and prevalent atherosclerotic vascular disease (ASVD) at baseline.

|  | **Quartiles of Vitamin K1**  ^1^ | | | |
| --- | --- | --- | --- | --- |
|  | **Quartile 1**  <61.6 ug/d | **Quartile 2**  61.6 to <79.0 ug/d | **Quartile 3**  79.0 to <99.2 ug/d | **Quartile 4**  ≥99.2 ug/d |
| **14.5 y any fall-related hospitalisation** |  |  |  |  |
| *Events, n (%)* | 148 (43.9) | 133 (39.5) | 133 (39.6) | 121 (35.9) |
| *Model 3* | Ref. | 0.86 (0.74-1.00) | **0.78 (0.64-0.93)** | **0.73 (0.58-0.93)** |
| *Model 3 + hand grip strength* | Ref. | 0.88 (0.76-1.03) | **0.79 (0.66-0.95)** | **0.75 (0.59-0.95)** |
| *Model 3 + timed-up-and-go* | Ref. | 0.89 (0.76-1.03) | **0.80 (0.67-0.97)** | **0.76 (0.60-0.96)** |
| *Model 3 + NRFI per 1000 kJ* | Ref. | **0.86 (0.74-0.99)** | **0.76 (0.63-0.92)** | **0.71 (0.55-0.92)** |
| *Model 3 + protein* | Ref. | **0.85 (0.73-0.99)** | **0.75 (0.62-0.93)** | **0.70 (0.53-0.93)** |
| *Model 3 + calcium* | Ref. | **0.85 (0.73-0.99)** | **0.75 (0.62-0.91)** | **0.70 (0.55-0.90)** |
| *Model 3 + Prevalent ASVD* | Ref. | **0.86 (0.74-0.99)** | **0.77 (0.64-0.93)** | **0.73 (0.58-0.93)** |

^1^Estimated hazard and 95%CI from Cox proportional hazards analysis comparing the median Vitamin K1 intake from each quartile (Q) compared to quartile 1. Median intake Q1, Q2, Q3 and Q4 for Vitamin K1 was 49.3, 70.1, 87.6 and 119.5 ug/d, respectively. Model 3: adjusted for age, treatment, body mass index, smoking history, physical activity, calcium, alcohol intake and prevalent falls, season and 25OHD. Bolded indicates p<0.05 compared to Q1.
